# Autism spectrum disorders: emerging mechanisms and mechanism-based treatment

**DOI:** 10.3389/fncel.2015.00183

**Published:** 2015-05-12

**Authors:** Hansen Wang, Laurie C. Doering

**Affiliations:** ^1^Faculty of Medicine, University of TorontoToronto, ON, Canada; ^2^Department of Pathology and Molecular Medicine, Faculty of Health Sciences, McMaster UniversityHamilton, ON, Canada

**Keywords:** autism spectrum disorders, fragile X syndrome, Rett syndrome, pathogenesis, treatment, synaptic deficits, FMRP, MeCP2

## Introduction

Autism spectrum disorders (ASDs) are a group of neurodevelopmental disorders characterized by impaired social communication, abnormal language development, restricted interests, and repetitive and stereotyped behaviors (Zoghbi and Bear, 2012; Ebert and Greenberg, 2013; Lai et al., 2014). These disorders show a high degree of clinical and genetic heterogeneity. Studies suggest that there is the functional convergence among autism-linked genes on common pathways that are involved in synaptic development, plasticity and signaling, raising the hope that similar therapeutic strategy may be effective for different forms of autistic disorders (Krumm et al., 2014; Ronemus et al., 2014). Investigation of cellular and synaptic deficits in ASDs will provide further insights into the pathogenesis of autism and may eventually lead to potential treatment for autism and other neurodevelopmental disorders (Zoghbi and Bear, 2012; Delorme et al., 2013; Ebert and Greenberg, 2013).

Our research topic entitled *Neural and Synaptic Defects in Autism Spectrum Disorders*, brings together 23 articles which document the recent development and ideas in the study of molecular/cellular mechanisms and treatment of ASDs, with an emphasis on syndromic disorders such as fragile X and Rett syndromes. In addition, model systems and methodological approaches with translational relevance to autism are covered in this research topic.

## Molecular, synaptic and cellular deficits in ASDs

Fragile X and Rett syndromes are leading the way in investigating the molecular mechanisms of autism (Krueger and Bear, 2011; Katz et al., 2012; Santoro et al., 2012). Fragile X mental retardation protein (FMRP) is an mRNA binding protein absent or mutated in fragile X syndrome (Bhakar et al., 2012; Santoro et al., 2012; Wang, 2015). *Westmark* highlights a study which demonstrated how FMRP cooperates with other autism-related molecules in experience-dependent synaptic pruning through proteasome-mediated degradation of postsynaptic density 95 (PSD-95) and how that mechanism fails in fragile X syndrome (Tsai et al., 2012; Westmark, 2013). FMRP interacts with other proteins, such as Slack channels and cytoplasmic FMRP interacting protein 1/2 (CYFIP1/2) (Pasciuto and Bagni, 2014). Abnormal Slack channel activity is implicated in fragile X syndrome. *Kim* and *Kaczmarek* describe the physiological role of Slack channels and how altered Slack channel activity leads to intellectual disability (Kim and Kaczmarek, 2014). *Abekhoukh* and *Bardoni* review the potential roles of CYFIP1/2 in intellectual disability and autism, and their relation to fragile X syndrome (Abekhoukh and Bardoni, 2014).

Rett syndrome is primarily caused by mutations in the methyl-CpG-binding protein 2 (*MECP2*) gene encoding the transcriptional repressor MeCP2 (Moretti and Zoghbi, 2006; Chahrour and Zoghbi, 2007). *Xu* and *Pozzo-Miller* comment on a study which identified a novel AT-hook domain of MeCP2 that plays important roles in chromatin organization, providing a mechanism that determines the clinical course of Rett syndrome and related disorders (Baker et al., 2013; Xu and Pozzo-Miller, 2013). The post-translational modifications of MeCP2 generate and regulate its functional versatility. *Bellini et al*. provide an overview of post-translational modifications as a mechanism for MeCP2 to control its involvement in synaptic plasticity and homeostasis (Bellini et al., 2014). The methyl-CpG-binding domain (MBD) of MeCP2 is crucial for its function as a transcriptional repressor. Zhao et al. provide further evidence from cultured hippocampal neurons and *in vivo* newborn neurons that mutations of MBD affect the roles of MeCP2 in neuronal development (Zhao et al., 2015).

Investigating the genes and genetic pathways involved in ASDs is essential to unraveling the pathogenesis of these disorders (Krumm et al., 2014; Ronemus et al., 2014). Banerjee et al. review how studies using animal models are providing key information for ASDs and discuss the genetic aspects of ASDs, emphasizing the conserved genes and genetic pathways implicated in autism (Banerjee et al., 2014). Chen et al. summarize the defects of synaptic proteins and receptors linked to ASDs and discuss their roles in the pathogenesis of ASDs via synaptic pathways (Chen et al., 2014).

Deficits in synapses and neural circuits underlie cognitive dysfunction in ASDs (Zoghbi and Bear, 2012; Ebert and Greenberg, 2013). Martin and Manzoni report that synaptic abnormalities persist into adulthood in the valproic acid rat model of autism and point out that the switch from hyper to hypo function in the medial prefrontal cortex might be related to neurodevelopmental defects in ASDs (Martin and Manzoni, 2014). Rotschafer and Razak review the auditory processing in fragile X syndrome, suggesting that auditory hypersensitivity could be a biomarker for fragile X syndrome and other ASDs (Rotschafer and Razak, 2014). Neuhofer et al. report on deficits in synaptic plasticity and dendritic spines within the nucleus accumbens of fragile X mice (Neuhofer et al., 2015). Doll and Broadie document the impairments in activity-dependent neural circuit assembly and refinement in ASD genetic models, particularly in the drosophila fragile X model (Doll and Broadie, 2014). Cea-Del Rio and Huntsman review how interneuron populations and inhibition contribute to the excitatory/inhibitory imbalance of neural networks in fragile X syndrome (Cea-Del Rio and Huntsman, 2014). Port et al. describe the convergence of circuit dysfunction in ASDs and discuss how studies focusing on neural circuit function help to identify common neurobiological mechanisms of ASDs (Port et al., 2014).

The advances in technical approaches and disease models have provided unprecedented opportunities to investigate neural and synaptic deficits in ASDs. In addition to mouse and rat models, other animals such as drosophila and C. elegans are now used to study autism (Doll and Broadie, 2014; Gamez-Del-Estal et al., 2014). The induced pluripotent stem cell (iPSC) technology combined with neural differentiation techniques allows detailed functional analysis of neurons generated from living individuals with neurological disorders (Bellin et al., 2012; Wang and Doering, 2012). In this research topic, Kim et al. summarize recent achievements in differentiating cortical neurons from human iPSCs and efforts to establish cell model systems to study ASDs using personalized neurons (Kim et al., 2014).

## Mechanism-based treatment

Pharmacological manipulation of neurotransmitter systems or signaling pathways linked to ASDs may provide therapeutic benefits for patients (Delorme et al., 2013; Ebert and Greenberg, 2013). Wang and Doering comment on a study which showed that targeting the downstream mTOR signaling pathway rectifies social behavior deficits in autistic mice (Gkogkas et al., 2013; Wang and Doering, 2013). The pharmacotherapy for fragile X and Rett syndromes is the focus of this research topic. The metabotropic glutamate receptor 5 (mGluR5) has been identified as a potential target for treating fragile X syndrome (Bhakar et al., 2012; Wang and Zhuo, 2012; Scharf et al., 2015). Gandhi et al. report that mGluR5 antagonist MPEP reverses maze learning and PSD-95 deficits in fragile X mice (Gandhi et al., 2014). The serotonin (5-HT) transporter inhibitor fluoxetine is prescribed for children with autism. Uutela et al. further document the behavioral and cellular responses to fluoxetine in the mouse model for fragile X syndrome (Uutela et al., 2014). Ciranna et al. review the potential therapeutic significance of 5-HT7 receptors for fragile X syndrome and other ASDs (Ciranna and Catania, 2014). Janc and Muller report that the free radical scavenger Trolox attenuates neuronal hyperexcitability, restores synaptic plasticity, and improves hypoxia tolerance in the hippocampal slices of *Mecp2*^−/*y*^ mice, suggesting that radical scavengers might be an option for treating neuronal dysfunction in Rett syndrome (Janc and Muller, 2014). Xu et al. report that the histone deacetylase-6 inhibitor Tubastatin-A improves BDNF trafficking in hippocampal neurons from *Mecp2* knockout mice, demonstrating that histone deacetylase-6 is a potential pharmacological target for treating Rett syndrome (Xu et al., 2014).

Lastly, Wang et al. provide a comprehensive review of current targeted pharmacological treatments for fragile X and Rett syndromes, and discuss related issues in both preclinical and clinical studies of potential therapies for ASDs (Wang et al., 2015). Since there are significant neurobiological overlaps among ASDs, the targeted treatments developed for fragile X and Rett syndromes will be highly relevant to other autistic disorders.

## Perspective

The increasing need for effective treatment of ASDs, together with the advancement of disease models and other technologies, are promoting studies toward identifying potential therapies. It is inspiring to see that research in animal models is translating into patients with ASDs. The successful development of mechanism-based treatment for autism will continuously require more extensive multidisciplinary collaboration among different research sectors (Katz et al., 2012; Delorme et al., 2013; Wang, 2014).

We thank the authors and reviewers for their efforts and hope that this research topic will enrich our knowledge of ASDs and spur new research interests in autism related biology.

### Conflict of interest statement

The authors declare that the research was conducted in the absence of any commercial or financial relationships that could be construed as a potential conflict of interest.
